# Assessment of the Fitbit Charge 2 for monitoring heart rate

**DOI:** 10.1371/journal.pone.0192691

**Published:** 2018-02-28

**Authors:** Simone Benedetto, Christian Caldato, Elia Bazzan, Darren C. Greenwood, Virginia Pensabene, Paolo Actis

**Affiliations:** 1 TSW XP Lab, Treviso, Italy; 2 Leeds Institute for Cardiovascular and Metabolic Medicine, University of Leeds, Leeds, United Kingdom; 3 Leeds Institute for Data Analytics, University of Leeds, Leeds, United Kingdom; 4 School of Electronic and Electrical Engineering, University of Leeds, Leeds, West Yorkshire, United Kingdom; 5 School of Medicine, Leeds Institute of Biomedical and Clinical Sciences, University of Leeds, Leeds, West Yorkshire, United Kingdom; University of Illinois at Urbana-Champaign, UNITED STATES

## Abstract

Fitness trackers are devices or applications for monitoring and tracking fitness-related metrics such as distance walked or run, calorie consumption, quality of sleep and heart rate. Since accurate heart rate monitoring is essential in fitness training, the objective of this study was to assess the accuracy and precision of the Fitbit Charge 2 for measuring heart rate with respect to a gold standard electrocardiograph. Fifteen healthy participants were asked to ride a stationary bike for 10 minutes and their heart rate was simultaneously recorded from each device. Results showed that the Fitbit Charge 2 underestimates the heart rate. Although the mean bias in measuring heart rate was a modest -5.9 bpm (95% CI: -6.1 to -5.6 bpm), the limits of agreement, which indicate the precision of individual measurements, between the Fitbit Charge 2 and criterion measure were wide (+16.8 to -28.5 bpm) indicating that an individual heart rate measure could plausibly be underestimated by almost 30 bpm.

## Introduction

Fitness trackers, also referred to as activity trackers, activity monitors or fitness bands are devices or applications for monitoring and tracking fitness-related metrics such as distance walked or run, calories consumed, and heart rate. The World Health Organization (WHO) recommends that adults aged 18–64 should do at least 150 minutes per week of moderate-intensity aerobic physical activity or at least 75 minutes of vigorous-intensity aerobic physical activity throughout the week (or a combination of both) to reduce the risk of chronic diseases and depression. Fitness trackers provide an easy interface for adults to meet those guidelines and, according to IDC worldwide quarterly device tracker, over 100 million units were sold in 2016. Fitness trackers, as the name suggests, have been conceived and marketed for tracking fitness related activities but, thanks to the continuous advancements in wearable technology, the potential applications have also expanded to include medical surveillance, non-invasive medical care, and mobile health-wellness monitoring [1, 2, 3].

Even if the reliability of wrist-worn trackers in clinical settings is still under debate, their adoption in human physiology research has been unanimously accepted in the last 2 years [1]. Consumer activity monitors, such as Fitbit, Apple watch, Jawbone, Microsoft band, to name a few, are now widely used in biomedical research to study therapeutic effects of self-monitoring, exercise therapy and behavioral interventions. An intense area of research aims at estimating the association between physical activity and metabolic function, cognitive and neurological health using consumer activity trackers. Also, interventional studies employed fitness trackers to improve the quality of life of breast cancer patients [https://clinicaltrials.gov/ct2/show/NCT02637765], and those who are giving up smoking [https://clinicaltrials.gov/ct2/show/NCT02422914]. The adoption of physical monitoring devices allowed scientists to identify specific biomarkers to predict lung function in young adults with asthma [NCT02556567] or surgical complications after abdominal cancer surgery [NCT02356471]. Finally, interventional studies support the value of wearable activity trackers as motivational tool for specific patients (e.g. for weight management in childhood obesity, diabetes, cystic fibrosis in adolescents, recovering alcoholics, peripheral artery disease, and knee osteoarthritis) and thus as a key factor for disease prevention and management.

Accurate and precise self-monitoring devices therefore provide potential benefit both to the patient, by providing real-time feedback on his specific physiological status, and to the health care provider, since they can collect and present a full set of information, including activity frequency, duration, and intensity, heart rate (HR), and energy consumption.

A key metric measured by fitness trackers is HR, namely the number of contractions of the heart per minute (bpm). Physical exercise, sleep, anxiety, stress, illness, and ingestion of drugs are all factors known to alter the normal HR and therefore HR has been used as an indicator of physiological adaptation and intensity of effort [4]. According to Takacs et al. [5], inaccurate measures of physical activity levels can affect the ability to monitor health status. Therefore, accurate HR monitoring is essential in fitness training and testing. Methods used to detect changes in HR include: electrocardiogram (ECG), blood pressure, ballistocardiograms and the pulse wave signal derived from a photoplethysmogram (PPG).

Recently, the need for affordable, simple and portable technology for both the primary care and community based clinical settings, together with the wide availability of low cost and small semiconductor components, have raised attention around PPG [6]. PPG is an optical measurement technique that measures the amount of backscattered infrared light through a tissue to assess the variation of blood volume and thus the heart rate [7]. According to Murthy et al. [8], photoplethysmography is a simple, reliable and low-cost optical technique for measuring changes in blood volume in the microvascular bed of tissue. Recent studies suggest this method has acceptable validity [9], although the accuracy is often dependent on the device used, the type and intensity of activity, and skin photosensitivity [10,11,12].

All wrist worn activity trackers rely on PPG and use proprietary HR-derived algorithms and several recent studies investigated the accuracy of wearable devices for measuring HR [10,13,14,15,16,17,18,19,20,21,22]. Here, we critically assessed the accuracy of the Fitbit Charge 2 (Fitbit Inc., San Francisco, CA) with respect to HR monitoring and compared its performance to a gold standard electrocardiograph. The Fitbit Charge 2 monitors HR activity—through a patented PPG technology called PurePulse—counts steps, calories burned and tracks sleep activity. Based on the manufacturer’s information, Fitbit uses PurePulse light-emitting diodes on the skin-facing surface monitor blood volume changes to continuously estimate HR [23]. In the past 2 years, several studies have been published on the most recent predecessor of this device, the Charge HR [10,13,14,16,17,18,20,21]. The large majority of these studies yielded similar results (i.e. aggregate mean biases between -2.5 bpm [21] and -9.3 bpm [10]). Since no studies have been published so far on the Charge 2, the purpose of our study was to evaluate for the first time the accuracy of this device for measuring HR with respect to an ECG criterion measure.

## Materials and methods

The accuracy of the Fitbit Charge 2 for measuring HR was assessed with respect to an ECG criterion measure (ProComp Infiniti T7500M). The ProComp Infiniti T7500M, is an 8 channel multi-modality encoder for real-time, computerized biofeedback and data acquisition in clinical setting (Thought Technology LTD, Toronto, CANADA). For ECG recording, the electrode placement sites were prepared by standardized procedures of cleaning, shaving, and abrading the skin to improve signal acquisition and to minimize noise artifact. Three silver/silver-chloride self-adhesive electrodes (RA, RL, LA) were placed on the upper torso. HR data per second was converted to beats per minute (bpm) automatically by the data acquisition software program prior to analysis. The Fitbit Charge 2 was placed on the non-dominant wrist following manufacturer instructions and was charged fully prior to testing.

Fifteen Caucasian participants (8 females, 7 males) took part in the experiment. Table 1 shows means, standard deviations (SD) and ranges for age, weight, height, and Body Mass Index (BMI). All participants gave written informed consent before participation. We excluded participants with neurological or cognitive disorders, recent musculoskeletal damage or surgery that would impair motor function, and tattoos. The study was performed in a controlled experiment room at TSW XP Lab, Treviso—Italy (www.tsw.it) complying with the Declaration of Helsinki. The TSW XP Lab Ethics Committee approved the study.

**Table 1:** Age, weight, height, and BMI means and ranges.

	Male		Female	
	Mean (*SD*)	Range	Mean (*SD*)	Range
**Age (Years)**	31 (*4*)	25 to 36	32 (*4*)	26 to 36
**Weight (kg)**	78 (*3*)	76 to 82	60 (*3*)	56 to 65
**Height (cm)**	180 (*3*)	175 to 185	165 (*5*)	155 to 175
**BMI (kg/m**^**2**^**)**	24 (*0*.*65*)	23 to 25	22 (1.22)	20 to 23

Standard deviations are presented in parentheses.

Participants were asked to ride a stationary bike with the stated goal to raise their HR as much as possible, but they were free to slow down and rest at any time they desired to do so. The goal of the experiment was not to evaluate the training activity but rather collecting enough HR data spanning a range of BPMs as wide as possible. HR was simultaneously acquired for 10 minutes using both devices (Fitbit Charge 2, ProComp Infiniti). Since at the time the experiment was conducted, the Fitbit Charge 2 did not allow the download of second-by-second HR data, HR information (i.e. bpm) provided by the official Fitbit app for Android was displayed on a dedicated smartphone and then recorded via an HD camera pointed towards the display. Second-by-second HR data was manually extracted from each video and subsequently used for analysis. During each recording, the correspondence between the information visualized on the app and on the Fitbit Charge 2 display was checked multiple times to ensure the reliability of data collection and no discrepancies were found. Agreement between the Fitbit device and the ECG gold standard was estimated using the Bland-Altman method, adapted to take into account repeated measures from the same person when the true value varies over time [24, 25]. This provided an estimate of agreement between the Fitbit and ECG in the instantaneous value of the changing heart rate. We also modelled the relationship between the paired differences and their average to assess the extent to which the agreement varied with heart rate, and estimated the intraclass correlation coefficient (ICC) as an alternative measure of agreement All statistical analyses were performed using Statsoft STATISTICA 10 and StataCorp Stata 15.1.

## Results

**Fig 1:**
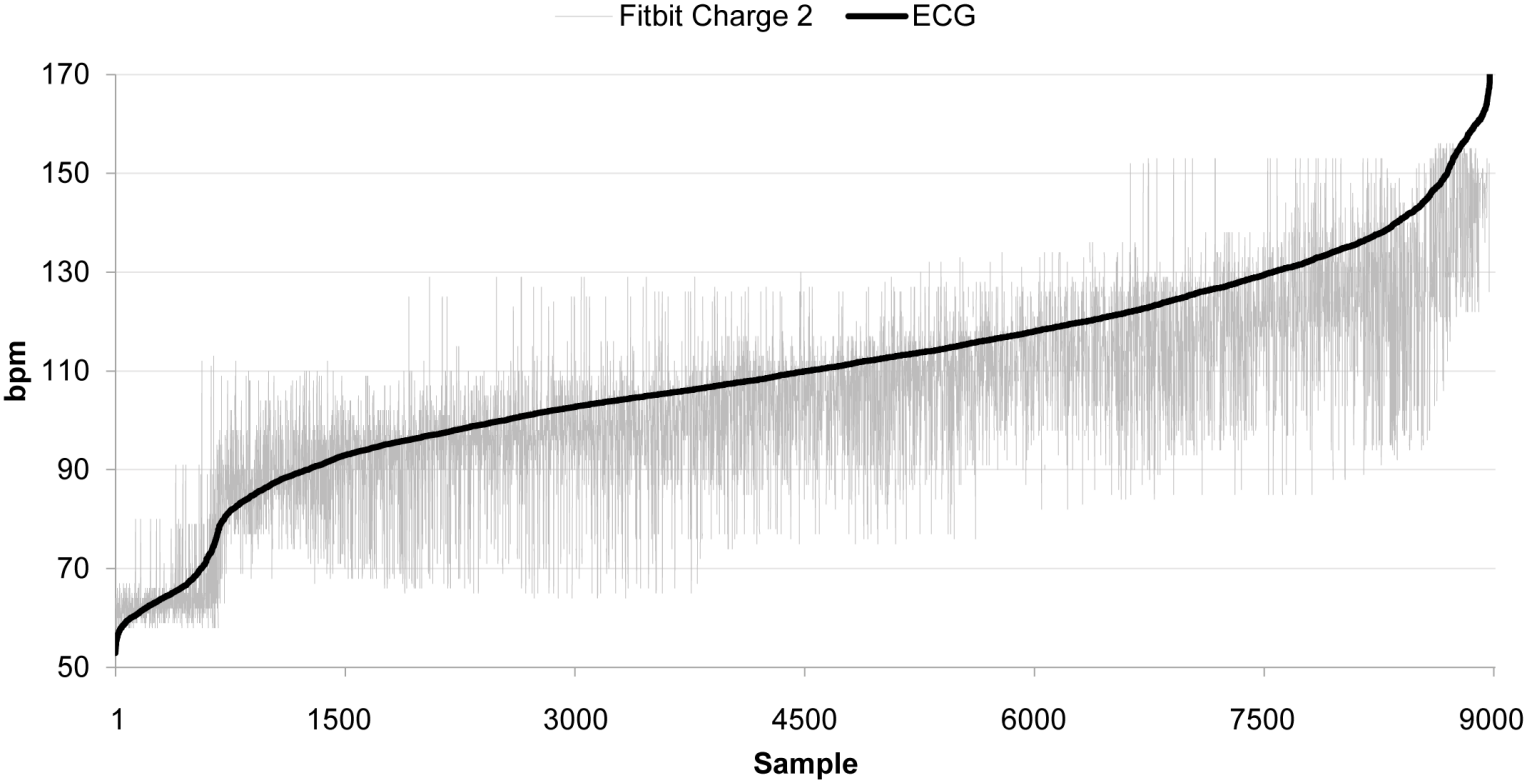
Ordered HR data (Fitbit Charge 2 vs. ECG). Data have been ordered according to the frequencies collected by the criterion measure (ECG). (n = 9000).

The dataset consisted of 9000 seconds of data (10 min x 15 participants). However, since the Charge 2 produced several disruptions to continuous HR detection, the dataset was reduced by around 10%. Fig 1. shows all time-synced ECG and Charge 2 HR ordered data in aggregate while Table 2 shows the HR comparison data between Charge 2 and gold standard ECG.

**Table 2:** Summary of HR comparison data between Charge 2 and ECG.

Parameter					
Fitbit Charge 2 Mean HR (bpm)	Gold Standard ECG Mean HR (bpm)	Mean Bias (bpm)	95% Upper LoA (bpm)	95% Lower (bpm)	Intraclass Correlation Coefficient (ICC)
102.7 (*20*.*1*)	109.8 (*20*.*9*)	-5.9 (*11*.*6*)	+16.8	-28.5	0.21

Standard deviations are presented in parentheses.

**Fig 2:**
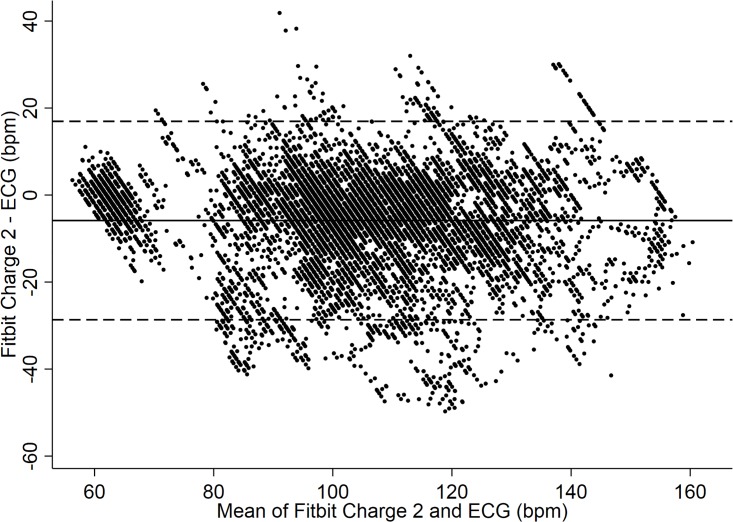
HR data (Fitbit Charge 2 vs. ECG). Bland-Altman Plot indicating mean difference in HR detection between the Charge 2 and ECG criterion measure. Mean bias and Limits of Agreement (95% LoA) are shown.

**Fig 3:**
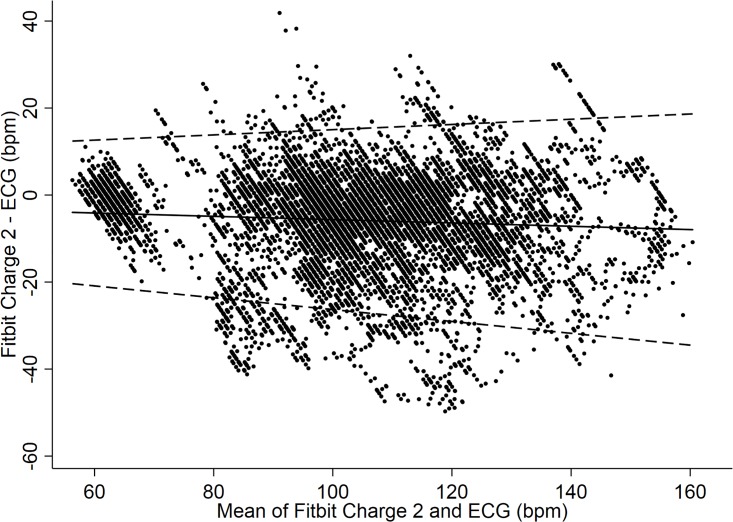
HR data with trend (Fitbit Charge 2 vs. ECG). Bland-Altman Plot modeling a trend over continuous heart rate indicating mean difference in HR detection between the Charge 2 and ECG criterion measure. Mean bias and Limits of Agreement (95% LoA) are shown.

The Charge 2 exhibited a mean bias of -5.9 bpm (95% CI: -6.1 to -5.6 bpm). As to the limits of agreement (LoA) between the Fitbit Charge 2 and criterion measure the upper LoA was +17 bpm, whereas the lower LoA was -29 bpm (Fig 2). The ICC between Fitbit Charge 2 and gold standard ECG was 0.21 (95% CI: 0.09 to 0.34). Furthermore, there was no evidence that the extent of agreement varied much across the range of heart rates (see Fig 3).

## Discussion

The aim of the present study was to assess in a controlled research environment the accuracy of the Fitbit Charge 2 for measuring HR. Our findings are in line with those of several recent publications involving the predecessor of this device (i.e. Fitbit Charge HR). Aggregate mean biases ranged from -2.5 bpm [21] to -9.3 bpm [10]. Wallen et al. [10], found the Fitbit Charge HR to have the highest bias among other three activity monitors tested (i.e. Apple Watch, Samsung Gear S, and Mio Alpha), reported an average error of -9.3 (± 8.5) bpm. Stahl et al. [16], and also found the Fitbit Charge HR to have the greatest bias among other five fitness trackers tested (Scosche Rhythm, Mio Alpha, TomTom Runner Cardio, Microsoft Band, Basis Peak). The criterion measure (i.e. Polar RS400) had a mean of 109.06 (± 29.3) bpm, whereas the compiled means of the Fitbit Charge HR was 105.00 (± 30.6) bpm. Other studies [13,17,18] fall within this range. The study by Cadmus-Bertram [21] was the one reporting the lower bias. We believe this might be imputable to the fact that participants exercised at just 65% of the maximum HR.

**Fig 4:**
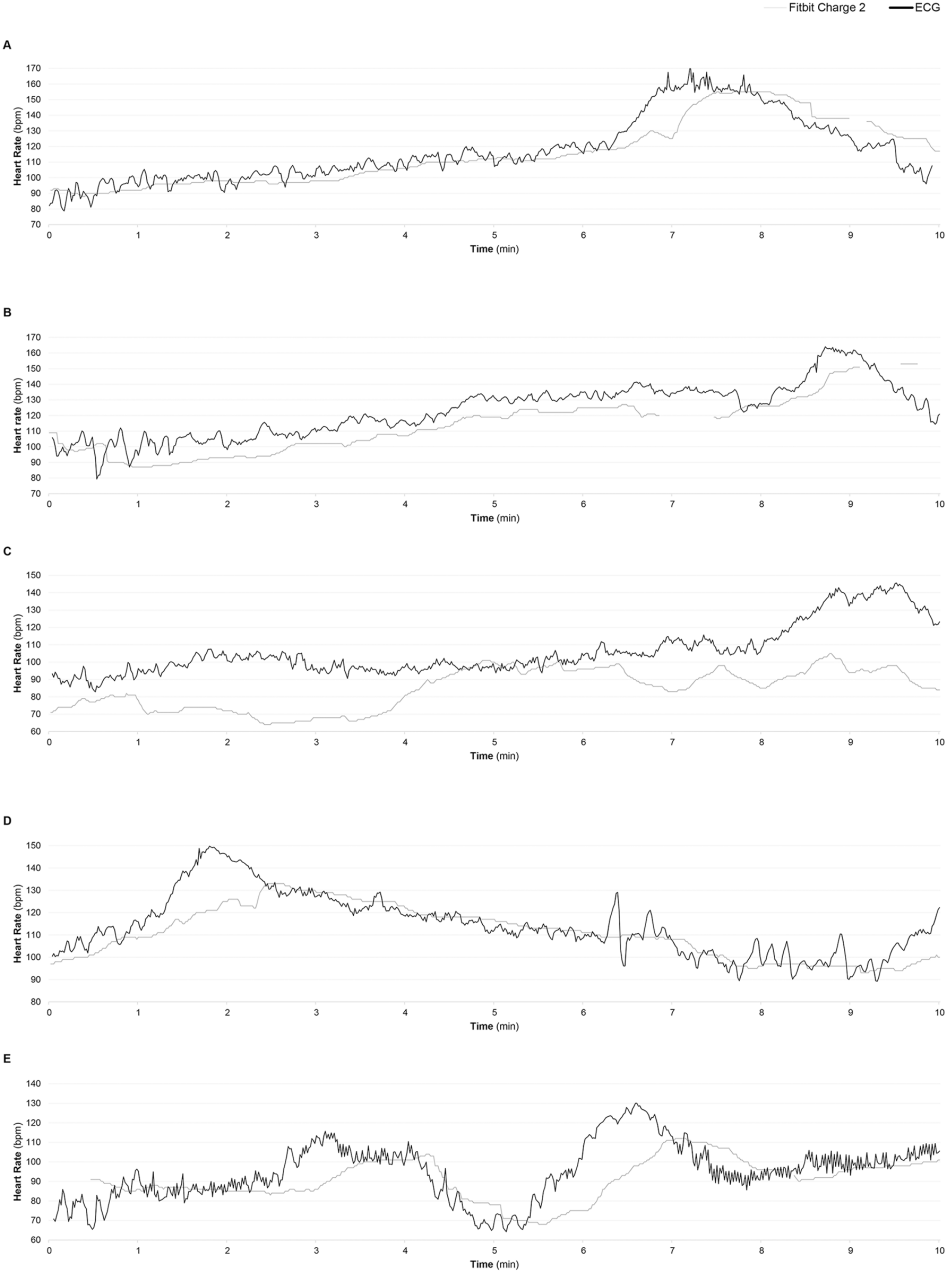
Representative time-series data of 5 participants (A, B, C, D, E).

Our results showed that the Charge 2 tends to underestimate the effective HR, with a bias and lack of precision that are fairly consistent across the range of heart rates. Since those inaccuracies occurred mainly during peaks of HR, it may be speculated that the current algorithms for HR estimation lack proper sophistication (Fig 4).

According to Takacs et al. [5], inaccurate measures of physical activity can affect the ability to monitor health status and are potentially dangerous for users. HR training relies in fact on exercising in different HR zones, each of which is a percentage of your maximum HR: once these are calculated, the workout can be tailored to the specific user, balancing fitness gain without overloading the heart activity.

Unstable positioning of the device, movement of the wrist compared to the rest of the arm and the whole body, variation in pressure of the sensor on the skin are the most plausible causes for this frequent problem for wrist-worn devices. Although our experimental setting was specifically built to control some of these issues, for 3 of 15 participants the Fitbit was not able to continuously register data (with a maximum of 35% of data loss).

Concerning the correlation between the position of the sensor and cycling activity, further studies need to be carried out to reveal if this new model of Fitbit is less accurate during cycling compared to walking or running activity. Studies performed on previous Fitbit models showed that the Fitbit Surge performed better during cycling than during high HR walking and running sessions [22], while they confirmed inaccurate performances of the Fitbit Charge HR during medium to higher intensity of activity, including low and intense cycling [13]. Since the specific algorithm used by the Fitbit is protected, it is not possible to explain the origin of the measurement discrepancies, and to discriminate if they are originated by software, firmware of hardware.

Our understanding is that intrinsic differences exist between PPG and ECG methods that calculate heart activity measuring different peaks and waveform of the heartbeat: specifically, by PPG in general, P peaks are recorded, and P-P intervals are evaluated without including high frequency components corresponding to heart beats; with an electrocardiogram instead, the full PQRST wave is registered, the interval between R-peaks are usually used to quantify the final HR. Inaccurate sampling and recording of the P peaks can definitely affect the final calculation of the HR by PPG. Furthermore, while we know that the gold standard instrument used in this study (i.e. PROCOMP Infiniti) extrapolates the complex HRV using an HRV resonant frequency detection, and calculates standard deviations and root mean square of R-R intervals, as well as the pNN50 values (i.e. percentage of pairs of adjacent P-P intervals differing by more than 50 ms), we do not know if the PurePulse technology relies on P-P or R-R intervals and how the pulse intervals are detected.

Future work will be devoted to a complete evaluation of new HR monitors performances and accuracy on a larger group of participants, including different skin characteristics, BMI and ages. Further studies will compare the Fitbit Charge 2 with previous models and equivalent wrist-worn devices on the market. The protocol for the evaluation of the performances will include a defined activity pattern for the participants in order to simulate low, medium and intensive exercise. The duration of these different training steps will allow the participant to keep a constant heart rate activity and breath rate for prolonged periods of time. This will allow the quantification of sampling efficiency for this technology, to identify strategies used for the representation of the data, and to distinguish algorithmic artifacts from unreliable data.

## Conclusions

The most recent Fitbit Charge 2 presents level of accuracy for HR measurement and performances unchanged from existing models of the same brand. Whilst there is only moderate bias on average, precision is poor for individual measurements, which could plausibly be underestimated by as much as 30 bpm. Although the exact algorithm used by the Fitbit device to estimate heart rate from the PPG measurements is not in the public domain, instability and improper positioning of the device may potentially explain different results and poor-quality HR data.

## Supporting information

S1 Dataset Study data. (XLSX)
